# Inclusive and Collaborative Exergame for Adults With Intellectual and Developmental Disabilities: Development and Usability Study

**DOI:** 10.2196/90156

**Published:** 2026-07-31

**Authors:** Chiara Piazzalunga, Samuele Ravazzani, Alberto Romano, Federica Maria Storti, Chiara Zelano, Emma Mencacci, Grazia Giana, Ottaviano Martinelli, Manuela Galli, Simona Ferrante

**Affiliations:** 1 Department of Electronics, Information and Bioengineering Politecnico di Milano Milan, Lombardy Italy; 2 Department of Mental Health and Addiction Alessandro Manzoni Hospital Lecco, Lombardy Italy; 3 LEARNLAB, Joint Research Platform Fondazione IRCCS Istituto Neurologico Carlo Besta Milan, Lombardy Italy

**Keywords:** co-design, collaborative, digital health, exergame, inclusive, intellectual and developmental disability, multiplayer, serious game, usability

## Abstract

**Background:**

People with intellectual and developmental disabilities (IDDs) face difficulties in being included in activities with their peers due to differences in cognitive abilities and social skills. Video games offer a promising medium to support inclusion, physical activity, and social engagement, but current solutions struggle to provide equitable experiences to heterogeneous groups of users, especially in multiplayer real-time contexts.

**Objective:**

This study aims to co-design and develop an inclusive collaborative real-time multiplayer exergame, assessing its usability, the impact of accessibility features, and players’ satisfaction and enjoyment.

**Methods:**

The exergame *Elemental* was co-designed following the CeHRes (Centre for eHealth and Wellbeing Research) Roadmap, involving clinicians, educators, engineers, and individuals with IDDs. A total of 2 cooperative minigames were developed: Igloo (focused on stimulus collection) and Volcano (focused on enhancing collaboration), playable with 4 different input devices (buttons, tablet, hand-tracking, and full-body tracking). Customizable facilitation options were implemented to adapt gameplay to sensory, cognitive, and motor needs. Young adults with IDDs participated in a 2-phase study testing whether personalized accommodations could eliminate performance disparities: (1) Igloo in homogeneous groups, based on functioning and expected behavior and interaction with stimuli, without facilitations, using all devices to identify optimal input methods, and (2) Volcano in heterogeneous groups using their best-performing devices with individualized facilitations tailored by educators. Data collected included in-game performance (accuracy, reaction time, and collaboration contributions), behavioral observations, and questionnaires on satisfaction and usability from players. Nonparametric analyses were used to assess relationships between disability severity, performance, and the impact of facilitations.

**Results:**

A total of 11 individuals (2 male and 9 female; mean age 25.1, SD 4.4 years) with different IDD diagnoses were recruited from an association supporting individuals with cognitive impairments. In the Igloo sessions, performance was negatively correlated with intellectual disability severity (ρ=–0.87, 95% CI –1.00 to –0.56; *P*<.001) and reaction time was positively correlated (ρ=0.69, 95% CI 0.08-0.94; *P*=.02). Instead, no significant correlation between performance and intellectual disability severity was observed in the Volcano sessions (ρ=0.24, 95% CI –0.48 to 0.79; *P*=.48). These results highlight that tailored support (personalized facilitations and best-suited devices) can foster equitable participation even in heterogeneous groups. Behavioral analysis revealed frequent peer collaboration. Participants reported high usability and satisfaction (median 4/5, IQR 0.5).

**Conclusions:**

This study introduces an inherently accessible, co-designed multiplayer exergame. Unlike approaches that adapt games or create separate disability-specific solutions, Elemental was conceived as inclusive from the outset. By demonstrating that personalized features can eliminate performance disparities, this work highlights how inclusive co-design can transform an activity into an inclusive, collaborative, and enjoyable experience for individuals with different abilities and intellectual impairments, supporting the shift from fitting individuals into existing digital spaces to designing environments able to embrace diversity.

## Introduction

### Problem

In recent years, there has been significant growth in awareness surrounding the needs of people with intellectual and developmental disabilities (IDDs) [1], but the attention of the scientific community is often directed toward diagnostics, etiology, and therapy [1,2]. While these are important, a broader perspective is needed that considers how people with IDDs experience daily life and participate in their communities. Building on the European Disability Strategy 2010-2020 [3], emphasis was placed on empowering people with disabilities to fully participate in society. Key objectives included independent living and community inclusion through “deinstitutionalization”—shifting from institutional care to community-based support that enables individuals to exercise choice and control over their lives.

This policy shift recognizes that people with IDDs often spend their time in group settings with peers and educators in day programs, schools, and residential facilities, where quality of life depends heavily on meaningful participation in shared activities, including play and recreation [4,5]. Within this framework, promoting autonomy and inclusion requires addressing not only environmental and social barriers but also developing essential life skills—emotional skills, social skills, and thinking skills—which are crucial for improving well-being, happiness, and interpersonal relations to facilitate full participation in community life [6].

Despite these policy commitments, people with IDD continue to face exclusion from leisure activities with their peers, with factors such as disability severity, environmental barriers, and societal attitudes exacerbating this phenomenon [7]. These barriers are especially difficult to eliminate when people with IDDs must engage in activities and tasks designed solely for neurotypical people, given that IDDs encompass a wide range of conditions with varying support needs, making each person’s requirements for meaningful participation quite different [8,9]. This diversity is especially important to consider in group settings where people with different abilities come together for shared activities, yet activities are typically designed without accounting for this individualized support emphasized in current inclusion frameworks [10].

Nevertheless, the social model of disability reminds us that all disabilities, including IDDs, stem not only from their underlying medical causes but also from the interaction between individuals and personal and environmental factors [11]. In group settings where people with IDDs spend much of their time, the design of activities, tools, and spaces should prioritize their needs, remove existing barriers, and prevent the creation of new ones [12]. When designing games, activities, and experiences for these environments, accessibility should be a crucial aspect to take into consideration. Accessibility means creating experiences that work for everyone in the group, regardless of different abilities and support needs. Therefore, accessibility is not only about adapting existing activities but about designing inclusive experiences from the start that allow everyone to participate meaningfully together [13].

Yet accessibility is not an easy feature to obtain, particularly when considering social contexts in which multiple individuals must interact. People with disabilities are drastically different from one another. Their needs may be complex, difficult to accommodate, or conflicting with the needs of another person when interacting in social contexts [14]. Although accessibility tools enable people with disabilities to use instruments and services, they often achieve this through extensive customization that creates separate experiences, potentially perpetuating feelings of exclusion. Thus, it is crucial to design and create an experience that is usable by all, while being perceived as fair and engaging.

### Review of Relevant Scholarship

Video games present a promising medium to address these challenges in group settings. They have shown positive effects on the quality of life of people with disabilities, providing entertainment opportunities, and have potential for inclusion through their ludic aspect that boosts motivation [15,16]. Moreover, they offer extreme customizability, making them particularly adequate to craft individualized experiences. The provided benefits are not limited to entertainment but encompass the possibility of providing therapeutic activities and social inclusion. Exergames, for example, are video games that combine gaming elements with physical activity, encouraging movement and exercise. These games can be useful for people with IDDs [17], as they offer a fun and engaging way to stay active, which is particularly important in a population that usually has lower physical activity levels [18]. Exergames can improve gross motor functioning and balance, contributing to an overall improvement in physical and mental health, while exploiting their fun side to motivate players [19].

Despite this potential, accessibility barriers in mainstream video games have made them almost unusable for people with disabilities, leading to their exclusion from this social phenomenon [20]. Lack of accessibility in a video game usually stems from the difficulty or impossibility of (1) capturing stimuli (sensory disability), (2) determining responses (cognitive disability), and (3) using input interfaces (motor disability) [21]. These issues are particularly problematic in multiplayer games, where several people participate simultaneously. Playing together requires that the games be accessible and that the players be prepared for different levels of ability, allowing all individuals to engage in the game at the same level of experience [22].

Encouragingly, the field of video games is recently becoming more and more accessible, with major companies such as Microsoft paying closer attention to all customers’ needs [23]. Efforts were made to develop or adapt video games to be accessible to various disabilities, including sensory, motor, and cognitive impairments, through software and hardware features.

Software ones are easier to implement and customize, alleviating barriers from various disabilities. For hearing impairments, subtitles are now widely adopted. Games such as Fortnite [24] and Minecraft [25] have added the possibility to display symbols or text accompanied by visual effects indicating the direction of an acoustic stimulus. Visual impairments are addressed in games such as The Last of Us [26], specifically in its second instalment, equipped with a text-to-speech system and audio pings that allow the player to locate enemies and loot. Software interventions allow for improved accessibility for people with cognitive disabilities as well. For example, Celeste [27] has an “Assist Mode” which allows for granular control of the game speed, delaying the reaction times necessary to make progress in the game.

From a hardware standpoint, to overcome motor and visual disabilities, controllers have instead been adapted to meet players’ needs. Guitar Hero was adapted for people with blindness (Blind Hero) [28], providing inputs through haptic stimuli via gloves rather than screen visuals. For limited mobility, Xbox has developed the “Xbox Adaptive Controller,” [29] a larger controller that can be customized with additional elements such as joysticks, buttons, and switches to meet players’ motor needs.

However, multiplayer games for people with IDDs remain understudied in literature and scarcely found in market solutions, as the challenge is not limited to making the games accessible, but to making the experience fair and enjoyable for everyone. Moreover, most current solutions lack a portfolio of settings that allows to create a truly customized experience.

### Hypothesis, Aims, and Objectives

Given these premises, the aims of this study are (1) to describe the co-design and development of an inclusive, collaborative, real-time multiplayer exergame; (2) to assess its usability with a group of individuals with IDDs through the analysis of players’ behavior; (3) to assess the impact of accessibility features on performance; and (4) to evaluate players’ satisfaction and enjoyment.

## Methods

This study combined a participatory co-design and development phase with a prospective 2-phase evaluation of the resulting exergame. The design was nonrandomized and observational with repeated measures across sessions.

### Co-Design and Development of the Elemental Game

#### Overview

During the creation of the game, particular care was devoted to the design process, as the users’ needs are very diverse and should be carefully considered to deliver an enjoyable experience. Consequently, the design process followed a pipeline inspired by the CeHRes (Centre for eHealth and Wellbeing Research) Roadmap 2.0 [30], a framework for the participatory development of eHealth technologies. The sequential steps of the CeHRes Roadmap are “Contextual Inquiry,” “Value Specification,” “Design,” “Operationalization,” and “Summative Evaluation,” although “Operationalization” was not included in this work, since it is a step that is better suited for commercial solutions.

#### Contextual Inquiry

This phase is crucial to lay down the foundations for the entirety of the design and development process. Its main goals are to identify the main actors involved in this study and the types of users of the game, to characterize the context in which the game would be used, and to analyze the main criticalities to be improved in the current context.

First, an internal brainstorming session with clinicians (neuropsychiatrists, psychologists, and psychomotor therapists), engineers (biomedical engineers and computer science engineers), and educational professionals (educators and teachers) was conducted to characterize the IDD population that would benefit from an inclusive exergame, following stakeholder identification best practices [31]. All professionals had expertise in the fields of inclusion, education, and eHealth, and were thus chosen because of their miscellaneous experiences with disability in various contexts.

The contexts in which the game would be used were analyzed through extensive literature research, with special attention to users’ daily lives and behaviors and to legislative and ethical issues.

Information gathered from stakeholders was then combined with scientific evidence to identify the main criticalities and pain points in the current solutions.

From this analysis, the target population was identified as adults with IDDs because of their experience in structured group settings. Adults with IDDs represent a particularly important population to address, as they face increasing challenges in accessing engaging and accessible activities as they age, while spending significant portions of their time in organized social environments where inclusive activities could have a substantial impact.

Through contextual analysis of the daily life of Italian adults with IDDs, it was found that they regularly participate in educational and recreational activities within contexts created by associations, day programs, and residential facilities, where they engage in group activities with the support of clinicians and educators. These structured social environments present an ideal setting for implementing inclusive gaming experiences, as they already facilitate group participation and have support staff present who can assist with activity facilitation. Furthermore, these contexts align with the deinstitutionalization principles emphasized in current disability policy, as they represent community-based settings where people with IDDs can develop social skills, engage in meaningful activities, and participate in shared experiences with their peers.

#### Value Specification

The discussion that originated during the “Contextual Inquiry” was crucial to refine the original, coarser idea of an inclusive exergame into a set of technical and functional specifications. Several needs emerged, which were then mapped to functional requirements, as seen in Textbox 1. Some functional requirements appear more than once, since they respond to more than one need.

**Needs and their correspondence to functional and technical requirements.**

**Customization**

- User profiles
- Possibility to tweak settings at runtime
- Multiple input devices

**Collaboration**

- Multiplayer game
- Real-time gameplay

**Accessibility**

- Multiple input devices
- Facilitations tailored for each need

**Inclusion**

- Facilitations tailored for each need
- Encouragement and rewards

**Clinically-informed design**

- Facilitations tailored for each need

**Engagement**

- Encouragement and rewards
- Possibility to tweak settings at runtime
- Attractive user interface
- Real-time gameplay

**Variety**

- Multiple games

**Cost-effectiveness**

- Reliance on inexpensive third-party services

**Cross-context deployment**

- Possibility to deploy on local servers

#### Game Design

##### Overview

Once the functional requirements of the game were defined, the design phase began. For each functionality, mock-ups were produced, analyzed, and discussed in multidisciplinary teams to assess the feasibility and adequacy of proposed solutions. After refinements, the technical team provided low-fidelity prototypes to be tested in a protected environment to identify any technical or usability issues. Finally, high-fidelity prototypes were tested by the main stakeholders to further refine the game.

The exergame developed was called Elemental and follows the daily activities of a little dragon named Blaze. Two minigames, the Igloo and the Volcano, were developed. In the Igloo, players should collect as many frozen objects as possible, whereas in the Volcano, all players must work together to guide Blaze, who is carrying stones, through a volcanic path toward the exit. The 2 minigames take place in the narrative framework of building a magical house for a farmer friend of Blaze, incorporating windows crafted from the ice collected and walls from the stones. This storyline was designed to promote altruism and reciprocal support among players.

The game uses a unique cooperative control system where each minigame requires players to manage distinct gameplay elements, making every participant essential to overall success while eliminating competitive dynamics. Although each player operates from their individual screen, all participants collectively control the same character, creating a truly collaborative gaming experience that emphasizes teamwork over individual achievement.

##### Facilitation Options

Facilitation options address multiple needs identified during the value proposition phase, particularly customization and accessibility. Since people with disabilities have vastly different needs, customization alone is insufficient: facilitation design must be guided by clinical evidence to effectively enable people with IDDs. These options were co-designed with clinicians to translate clinical requirements into implementable technical specifications (Table 1). Moreover, the approach to their definition should not rely too heavily on diagnoses, but on specific needs, as generalization is harmful not only across diagnoses, but across individuals with the same diagnosis as well. Because of this reason, some facilitation options were grouped together as presets, allowing for a quick selection, but there is always the option to toggle individual settings as well, to cater to every case.

**Table 1:** Definition of clinical requirements and their translation into functional requirements and practical game implementations.

Impairment	Clinical requirement	Functional requirement	Game implementation of the facilitator
Cognitive and sensory	Emphasizing one’s own task	Enhanced input feedback and action clarity	Reducing the visibility of other players’ stimuli No distractors Absence of stimuli requiring an inhibition response
Cognitive	Frequency modulation of stimuli	Adjustable game parameters	Current player’s stimuli appear less often
Cognitive and physical	Assisted action	Additional reference on the screen and compensatory reaction	3D model that mimics the actions that the player is supposed to do Automatic progression after long pauses
Sensory	Reduced audio stimuli	Reduced volume of music and sound effects	Reduced sound
Physical	Reduced movements	Reduction of frequency and range of motion	Current player needs to move less often and with smaller movements Player can play while seated
Sensory	Visual contrast modulation	Visual emphasis of important game elements	Increased contrast

Further images of the facilitation options visible on screen are available in Multimedia Appendix 1.

##### Instrumentation

A total of 4 distinct input systems were integrated to allow for a broader range of interaction modalities with the game:

- Cosmo Switch Buttons by Filisia [32], which are multicolored Bluetooth accessibility buttons that the user could push with their hands to control game actions.
- An iPad tablet by Apple [33], whose gyroscope was leveraged for a motion-based interaction. Users could hold the device with both hands on opposite sides and tilt it to control the interaction.
- The Leap Motion Controller by Ultraleap [34], which allows hand tracking. This device allowed users to interact with the game by moving their hands, without holding physical controllers; instead, a virtual representation of the hands interacted with virtual targets.
- The Homing system by Tecnobody [35], a small PC that uses an RGB-D camera to provide full-body tracking. This system allowed user to move freely and interact with virtual targets through a virtual representation of their body.

These devices require varying ability levels, meaning the motor and cognitive effort needed to interact with the game differs across input methods. Each device demands different combinations of cognitive and motor skills, which enhance the exergame’s accessibility and adaptability. This approach ensures that players with diverse needs and capabilities can find suitable ways to participate, creating a more inclusive gaming experience.

##### Architecture

#### Overview

The technical architecture of the platform was designed to support real-time multiplayer gameplay with extensive customization capabilities and future extensibility. The system consists of a Unity-based game client and a dedicated game server, both architected to accommodate variable numbers of players, multiple input devices, and individualized user profiles with tailored facilitations.

#### Client

The game client was developed using Unity 2022.3.10f1. As a real-time multiplayer platform consisting of several minigames, special attention was devoted to its code structure and architectural design. The design prioritizes modularity, particularly given that the game supports different input devices that can assume any role and accommodates a variable number of players per match. Furthermore, since the platform is designed with the future addition of new minigames and features in mind, extensibility was a key consideration throughout development.

#### Server

For the multiplayer game server, the main requirements considered were real-time functionality as well as turn-based multiplayer support. The service needed to support user profiles and authentication, enabling caregivers to log in and select individual profiles. Each profile maintains separate settings and facilitations, game points, and progression data, all of which must be centrally manageable by the caregiver.

A central control dashboard was required for the platform, accessible via the internet to allow teachers to edit student profiles. Additional requirements included minimal dependency on third-party online services, deployability on local servers, customization capabilities via scripts, and cost-effectiveness.

Given these comprehensive requirements, Nakama Game Server was selected as the optimal solution. The game server uses a CockroachDB instance as its primary database, where both game and analytical data are stored.

#### The Elemental Game

##### The Igloo Game

**Figure 1:**
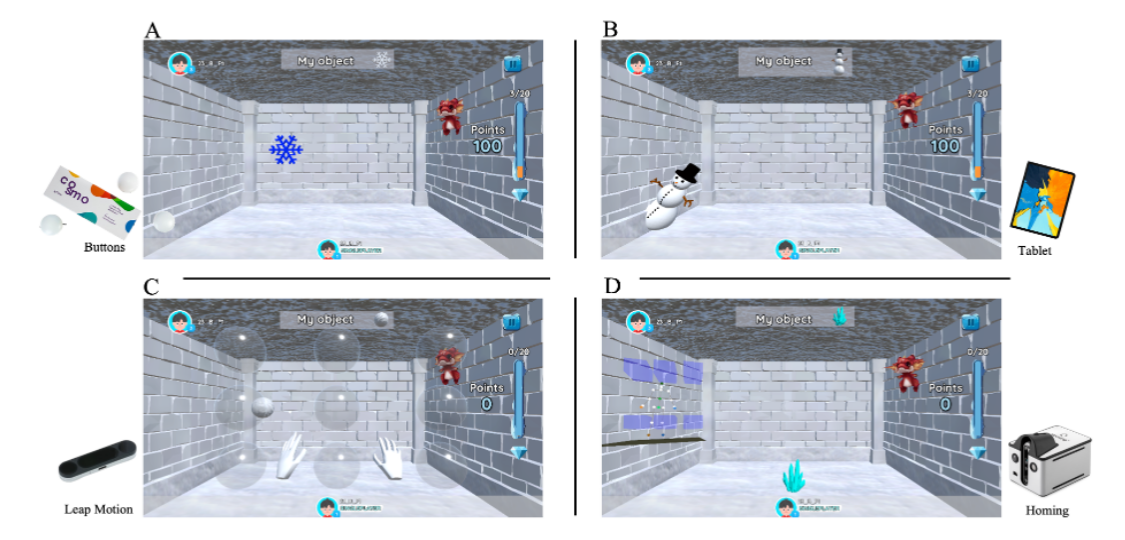
The user interface for each input device in the Igloo minigame. (A) The buttons and the snowflakes they have to collect, (B) the tablet and the snowman that must be collected by tilting the tablet, (C) the Leap Motion Controller with the virtual hands guided by the player’s hands that can collect the snowballs when interacting with the virtual bubbles, and (D) the Homing which presents a virtual representation of the player’s skeleton and the crystal they can collect.

The first minigame is the Igloo minigame. The stimuli presented are objects thematically connected to ice and snow. The stimuli appear on the screen for a fixed amount of time, and each player must interact with their input device to collect the object before it disappears. Table 2 contains the presented stimuli, the device associated with each of them, and the movement that needs to be carried out to collect the object. Examples of the different interfaces can be seen in Figure 1.

**Table 2:** Correspondence between input devices, stimuli, and requested movement.

Device	Object	Movement
Cosmo Buttons	Colored snowflakes	Push the button with the same color as the snowflake on the screen
Tablet	Snowmen	Tilt the tablet rightwards or leftwards
Leap Motion	Snowballs	Position the hands to intercept the snowballs
Homing	Crystals	Depending on where the crystals appear, position oneself to the left, center, or right, then jump or squat

The focus of this game is the collection of predefined appearing stimuli, which must be followed by a reaction of the player to collect their target stimulus. Its structured and stimulus design makes it well suited for comparing performance across different input devices and for collecting reaction time data. The setting is as simple as possible, and the only mechanic is the one that allows the player to collect the object. Moreover, to assess and, eventually, train inhibition, random red stimuli appear. These stimuli are distractors, and players should ignore them.

Several game parameters can be adjusted:

- The presence of red distractors.
- The number of stimuli on the screen at the same time.
- The total number of objects that appear in a match.
- The duration of each stimulus.

##### The Volcano Game

The Volcano minigame challenges players to guide Blaze safely out of a volcano following a path before rising lava reaches him, while also clearing the fog that gradually obscures the screen, hindering the visibility of all the players.

Each player controls Blaze’s movement in 1 of 4 directions (top, bottom, left, and right) and is called to perform the correct movement depending on their input device when Blaze arrives at a point in the path in which he needs to go in that direction. The direction is randomly assigned at the beginning of the match, and the path is procedurally generated to make sure that the path is navigable given the number of players and to ensure that everyone plays a crucial role in successfully leading Blaze to the exit.

The main aim of the Volcano minigame is collaboration, so a crucial mechanic is suggestions. When it is not their turn, players can still perform their own movements, signaling the direction they think the active player should go. The active player will see the suggestions on their own screen.

Another collaboration mechanic is the fog. During the entirety of the match, the screen will become less and less visible due to the fog produced by the volcano. To clear it, players must perform other movements, generally more physically demanding. The removal of the fog is much faster if all players contribute to it.

**Figure 2:**
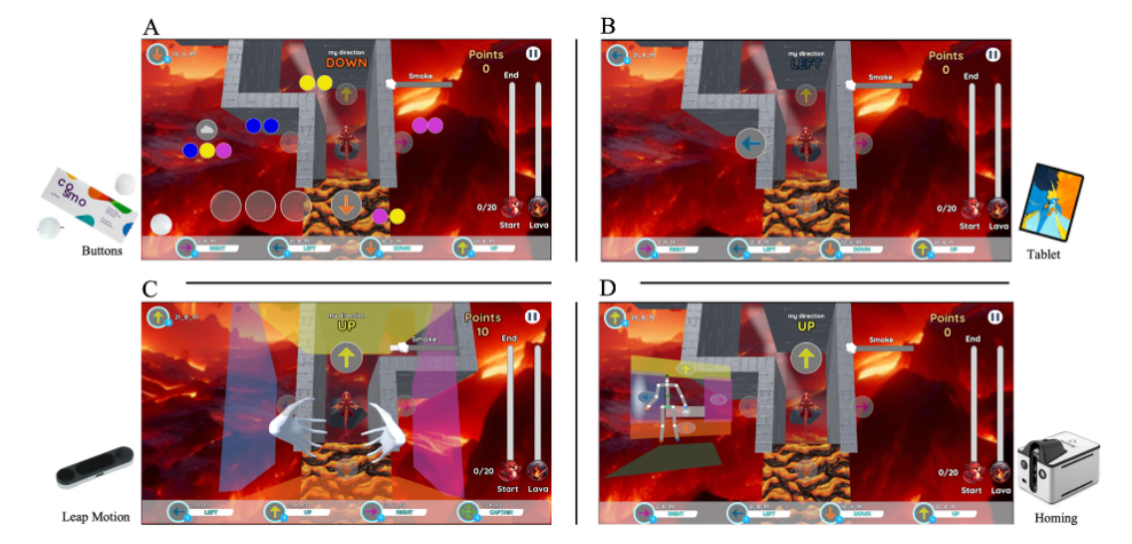
The user interface for each input device in the Volcano minigame. (A) The buttons with a sequence corresponding to each action, (B) the tablet that must be tilted, (C) the Leap Motion with the virtual hands that must collide with virtual walls, and (D) the Homing with a virtual skeleton and targets to be touched in order to perform the corresponding action.

Table 3 contains the input devices and, for each of them, the movements requested to move Blaze along the path and to remove the fog. Examples of the different interfaces can be seen in Figure 2.

Several game parameters can be adjusted:

- The length of the path.
- The speed of the lava.
- The intensity of the fog.

**Table 3:** Correspondence between input devices and requested movements for path progression and for fog removal.

Device	Movement for path progression	Movement for fog removal
Cosmo Buttons	Push the buttons in a predetermined sequence, one for each direction (shown on screen)	Push the buttons in a predetermined sequence (shown on screen)
Tablet	Tilt the tablet toward the desired direction	Shake the tablet horizontally
Leap Motion	Move both hands toward the desired direction	Shake hands vertically
Homing	Move both hands toward the desired direction	High knee running

#### Testing

In this phase, the game was tested on its final users to assess its usability, accessibility, and players’ satisfaction and enjoyment.

#### Inclusion and Exclusion

Inclusion criteria were being a young adult with IDD attending the collaborating association and being able to participate in the testing sessions. The exclusion criteria were complete blindness, contraindication for immersive technologies (eg, photosensitive epilepsy), and conditions causing loss of voluntary hand movement (eg, tetraplegia).

#### Participant Characteristics

The severity of the IDD among recruited participants was classified by one of the educators that follows them during the association’s activities into 4 categories: “mild,” “moderate,” “severe,” or “profound,” based on their intellectual functioning and adaptive behavior. The educators who provided the severity classification regularly worked with the participants during the association’s activities and familiarized them with the game context through narration, photos, and videos. This process allowed participants to be gently introduced to the experience and enabled a semiquantitative evaluation of their functional abilities relevant to the exergame.

#### Sampling Procedures

Young adults with IDD were recruited from the Associazione per lo Sviluppo del Potenziale Cognitivo (ASPOC) in Lecco (Italy) [36], a parent-led association supporting individuals with cognitive impairments. Recruitment followed a convenience sampling approach, based on the availability of eligible members and agreement from educators and legal guardians. Data collection took place at the Polo territoriale di Lecco of Politecnico di Milano, with 1 session per week over consecutive weeks between October and November 2024. No monetary compensation was provided.

#### Sample Size, Power, and Precision

No formal a priori power analysis was conducted because the study aimed primarily to evaluate usability, accessibility, players’ satisfaction and enjoyment, and preliminary performance patterns in a real-world group setting.

#### Conditions and Design

**Figure 3:**
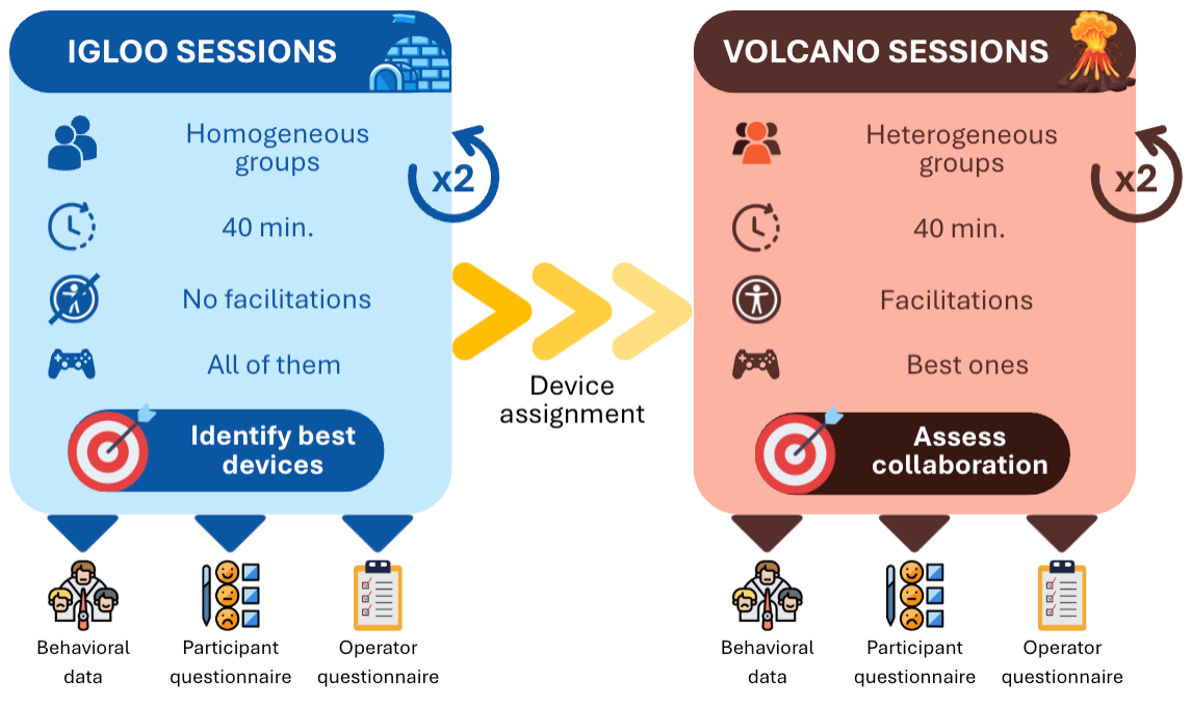
Protocol scheme overview. The protocol included 2 Igloo sessions (homogeneous groups, no facilitations, 40 minutes each) to identify optimal devices, followed by 2 Volcano sessions (heterogeneous groups, personalized facilitations, 40 minutes each) to assess collaboration. Behavioral data and questionnaires (participant and operator) were collected after each session.

The protocol, represented in Figure 3, consisted of 4 total 40-minute testing sessions. During each testing session, an operator was available for each participant to explain gameplay and interaction details and to support them in case of difficulties.

The protocol started with 2 sessions devoted to the Igloo minigame. Considering that the Igloo minigame is designed to collect appearing stimuli, participants were divided into homogeneous groups, that is, groups included individuals with comparable functioning, expected behavioral and interaction with game stimuli, according to the qualitative assessment of the educator in order to ensure a smooth gameplay experience for all group players. To evaluate their initial interaction with the technology, each participant was supposed to play this minigame using all devices, with no facilitation options and with fixed settings. A first match served as a familiarization phase, followed by a standard trial in which each player had to collect 5 stimuli, each appearing one at a time and requiring a reaction within 10 seconds.

Device performance accuracy was analyzed to define 2 pairs of input modalities (higher- and lower-performing). For each participant, mean accuracy was calculated for each device pair. Within each Volcano minigame group, participants were ordered by intellectual disability severity, with ties resolved by prioritizing those with lower cumulative mean accuracy. Participants were then assigned to the device pair yielding higher mean accuracy, subject to logistical constraints in device availability and balanced group allocation. The Volcano minigame was then played in heterogeneous groups, combining individuals with varying technological proficiency to encourage reciprocal help.

All participants used facilitation options tailored to their individual needs as determined by their educator. Assignment criteria included:

- Clearer input instructions: for those who require an appropriate stimulus but can be easily distracted.
- No distracting stimuli: for those who experience performance anxiety, helping them focus on essential gameplay elements without further distractions.
- 3D model advisor: for players who can imitate movements or those who usually try to click on the screen instead of moving.
- Auto-go: for players with severe difficulties in initiating movements, particular difficulties with a device, extreme performance anxiety, or extremely slow movements.
- Seated mode: for those who present mobility impairments.
- Less movement: for those who may encounter difficulties in understanding movement-based tasks and in executing physical actions.
- High contrast: for players with visual impairments.

Due to the modular nature of the facilitation system, each participant had the possibility to have more than one facilitation option active at a time.

#### Measures and Covariates

Since the aims of this work were both to assess the impact of accessibility features on performance and to evaluate usability and players’ satisfaction and enjoyment, the collected data can be categorized into 2 groups: game performance data and behavioral, satisfaction, and usability data. The first group includes in-game indicators recorded by the game platform, while the second comprises data obtained from behavioral observations of operators and ad hoc questionnaires completed by operators and participants, as described in the following paragraphs.

Intellectual disability severity was the main participant-level variable examined in relation to performance outcomes. In addition, device assignment, facilitation options, group composition, and minigame condition were considered relevant contextual factors when interpreting performance outcomes.

#### Data Collection

##### Game Performance Data

Several performance indicators were collected within each minigame. In the Igloo minigame, the main indicators collected were accuracy and reaction time. Accuracy was defined as the ratio between the number of objects successfully collected and the total number of objects available, considering only the second match since the first match served as familiarization. Reaction time was defined as the time elapsed between the appearance of the stimulus on the screen and the moment when the player collected the stimulus.

In the Volcano minigame, two main metrics were collected: accuracy and contributions to fog removal. Accuracy was defined as the ratio between the number of movements each player made for path progression (excluding clearing fog movements) during their designated turn and the total number of path-progression movements they made, considering both designated and nondesignated turns. Contribution to fog removal was measured as the number of times each player spontaneously performed the expected movement for the assigned device to clear the fog.

##### Behavioral, Satisfaction, and Usability Data

To assess the overall game experience, one operator collected the participant behaviors that emerged during gameplay. Each observed behavior was categorized along 3 dimensions: emotional valence (positive/negative), mode of expression (verbal/nonverbal), and social context (individual/social).

At the end of each session, operators present at each station completed an ad hoc questionnaire designed to assess usability and the ease of use of the technology for each player. This operator questionnaire was adapted from relevant items of the *International Classification of Functioning, Disability, and Health*, and consisted of 6 questions:

- Q1: Did the player interact with technology/experience in an accepting rather than oppositional way?
- Q2: Was the player able to use the technology by understanding its requirements and actively participating in the activity?
- Q3: Was the player able to control the movements required to perform the technology-based activity?
- Q4: Did the player demonstrate appropriate behavioral and emotional regulation during the activity?
- Q5: Did the player interact with others in a contextually and socially appropriate way?
- Q6: Did the player engage with others to achieve the goal of the activity? (Volcano only)

Questions were answered on a 5-point Likert scale, with response options ranging from 1 (“not at all”) to 5 (“a lot”): not at all, no, fairly, yes, and a lot. The first 3 questions were repeated for each device, while the latter 3 were compiled once for each testing session.

Finally, to collect players’ satisfaction and their opinion regarding the usability of the system, an ad hoc questionnaire was used:

- Q1: Did you enjoy playing this game?
- Q2: Would you like to play this game again?
- Q3: Did you understand how to play?
- Q4: Is it easy to play this game?
- Q5: Did you feel good at it?
- Q6: Did you play with your teammates? (Volcano only)

Questions were answered on a 5-point Likert scale, with response options ranging from 1 (“not at all”) to 5 (“a lot”): not at all, no, so and so, yes, and a lot. The augmentative and alternative communication was used to increase the accessibility of the questionnaire. This questionnaire was administered to each participant at the end of each session.

#### Quality of Measurements

To enhance the quality of measurements, a familiarization match was performed before the standard trial of the Igloo minigame, and an operator was present at each station during testing to explain gameplay and support participants in case of difficulties. Behavioral observations were recorded during gameplay using predefined categories derived from the co-design phase. Additionally, augmentative and alternative communication was used to increase the accessibility of the questionnaires.

#### Analytic Strategy

##### Game Performance Data

The analysis aimed to investigate the presence of a relationship between intellectual disability severity and gaming performance and, if present, to test whether personalized facilitations adopted during gameplay could mitigate it.

Indeed, it was hypothesized that the Igloo minigame, played without facilitations and using all available devices, would show a significant inverse correlation between disability severity and performance, while the Volcano minigame, played with personalized facilitations and optimal device assignments, should not show such a relationship. Given the limited sample size, nonparametric statistical methods were used throughout the analysis. Data were analyzed in Python 3.9 using medians and IQRs both at the individual level and grouped by device. To examine the association between disability severity and game performance, Spearman rank correlation (α=.05) analyses were conducted. Specifically, correlations were computed between intellectual disability severity scores and individual median accuracy values for both minigames. For the Igloo minigame, additional analyses were performed to assess the correlation between intellectual disability severity scores and individual reaction times.

##### Behavioral, Satisfaction, and Usability Data

Behavioral observations were analyzed using frequency counts for each behavior category.

Data from the operator questionnaire were analyzed using the median and IQR between participants for each minigame, question, and device. For the answers regarding sessions in which the participants used the same devices, the median response was considered.

Concerning the satisfaction and usability questionnaire, for each minigame and each question, the median and IQRs were computed across sessions and participants.

#### Ethical Considerations

This study was conducted in accordance with a protocol approved by the Politecnico di Milano Ethics Committee (approval number 19/2023). Given that all participants had IDD, informed consent was obtained from their legal guardians prior to participation. Researchers explained the project to participants to ensure their understanding of the study procedures and their right to withdraw at any time.

All participant data were pseudonymized to protect privacy and confidentiality. Participant identifiers were stored in a separate database from gameplay and behavioral data, with access restricted to authorized research personnel only. The linkage between personal information and pseudonymized codes was maintained securely and separately from the analytical dataset.

Participants received no monetary compensation for their involvement in the study. However, all activities were designed to be engaging and enjoyable, with breaks provided as needed.

No identifiable participants appear in any figures or videos in the main manuscript. The supplementary materials include video demonstrations of gameplay; however, these feature researchers rather than study participants, ensuring that no participant identification is possible.

## Results

### The Elemental Game

Videos showing player interactions with the game devices and photos of the facilitation options used during the testing sessions are available in Multimedia Appendix 2.

### Recruitment

Table 4 contains information about participants, including sex, age (mean 25.1, SD 4.4 years), diagnosis, and the disability level they were classified into.

**Table 4:** Information about the participants, including diagnosis, sex, age, and intellectual disability level, as defined by their educator.

Participant	Diagnosis	Sex	Age (years)	Intellectual disability level
001	Down syndrome	F	21	Severe
002	Down syndrome	F	22	Mild
003	Down syndrome	F	36	Moderate
004	Coffin-Lowry syndrome	M	26	Profound
005	ASD^a^ and Down syndrome	F	19	Mild
006	Turner syndrome	F	24	Mild
007	ZTTK^b^ syndrome and ADHD^c^	M	26	Severe
008	Intellectual disability	F	24	Mild
009	Down syndrome	F	25	Moderate
010	ASD and intellectual disability	F	28	Moderate
011	Intellectual disability	F	25	Mild

^a^ASD: autism spectrum disorder.

^b^ZTTK: Zhu-Tokita-Takenouchi-Kim.

^c^ADHD: attention deficit/hyperactivity disorder.

### Statistics and Data Analysis

#### Game Performance Analysis

##### Igloo Minigame

Table 5 displays for every participant the group to which they were assigned, their intellectual disability severity, median accuracy obtained for every device, and their general median accuracy across all devices. Although groups included participants with varying disability severity levels, their functioning and expected behavior and interaction with game stimuli were considered sufficiently comparable for the purposes of the Igloo game. This decision was made to ensure a smooth and coherent gameplay experience for all participants, even when groups were heterogeneous in other aspects. Missing data occurred for participant 010 (moderate intellectual disability severity), who did not use the tablet and Leap Motion devices, and participant 011 (mild intellectual disability severity), who did not use the tablet and Homing devices in the Igloo minigame, due to unexpected unavailability during scheduled sessions.

**Table 5:** Participants’ intellectual disability level and their Igloo minigame performance data.

Group and participant			Intellectual disability severity		Reaction time (seconds), median (IQR)										
					Buttons		Tablet		Leap Motion		Homing	Overall			
I1															
	001	Severe		1.00^a^ (0.00)		0.20^a^ (0.30)		0.40 (0.20)		0.40 (0.20)		0.60 (0.60)			
	002	Mild		1.00 (0.00)		1.00 (0.00)		1.00^a^ (0.00)		0.50^a^ (0.10)		1.00 (0.20)			
	003	Moderate		1.00^a^ (0.00)		1.00^a^ (0.00)		1.00 (0.00)		0.40 (0.10)		1.00 (0.60)			
	004	Profound		1.00^a^ (0.10)		0.70^a^ (0.30)		0.40 (0.00)		0.30 (0.10)		0.40 (0.60)			
I2															
	005	Mild		1.00^a^ (0.00)		1.00^a^ (0.00)		1.00 (0.00)		0.70 (0.10)		1.00 (0.00)			
	006	Mild		1.00 (0.00)		1.00 (0.00)		0.90^a^ (0.10)		0.90^a^ (0.25)		1.00 (0.15)			
	007	Severe		0.90^a^ (0.35)		1.00^a^ (0.00)		0.70 (0.10)		0.20 (0.00)		0.80 (0.55)			
	008	Mild		1.00 (0.00)		0.80 (0.20)		1.00^a^ (0.00)		0.80^a^ (0.10)		1.00 (0.10)			
I3															
	009	Moderate		1.00 (0.00)		1.00 (0.00)		1.00^a^ (0.00)		0.80^a^ (0.05)		1.00 (0.10)			
	010	Moderate		1.00^a^ (0.00)		—^a,b^		—		0.80 (0.40)		0.90 (0.20)			
	011	Mild		1.00 (0.00)		—		1.00^a^ (0.00)		—^a^		1.00 (0.00)			

^a^The device was later assigned to the Volcano minigame.

^b^Not available.

Table 6 displays for every participant the group to which they were assigned, their intellectual disability severity, median reaction time obtained for every device, and their overall median reaction time across all devices.

**Table 6:** Participants’ intellectual disability level and their Igloo minigame reaction time data.

Group and participant		Intellectual disability severity	Accuracy, median (IQR)				
			Buttons	Tablet	Leap Motion	Homing	Overall
I1							
	001	Severe	5.40 (1.38)	8.03 (2.33)	6.55 (4.20)	5.30 (4.38)	5.57 (2.43)
	002	Mild	1.69 (0.99)	3.21 (3.25)	2.90 (6.43)	5.08 (1.60)	2.65 (4.29)
	003	Moderate	4.38 (1.36)	5.45 (2.11)	7.09 (7.06)	6.98 (1.83)	5.11 (3.16)
	004	Profound	7.52 (3.85)	7.01 (1.80)	4.00 (3.67)	9.53 (2.58)	7.32 (3.17)
I2							
	005	Mild	2.53 (0.99)	2.24 (1.36)	3.03 (2.05)	9.30 (4.52)	2.77 (2.38)
	006	Mild	1.85 (0.67)	3.59 (1.89)	2.12 (3.67)	4.47 (1.17)	3.59 (2.81)
	007	Severe	4.50 (1.98)	5.34 (2.59)	4.03 (5.21)	9.19 (2.59)	4.92 (2.84)
	008	Mild	1.65 (0.25)	4.89 (1.37)	1.63 (0.58)	4.70 (2.22)	2.03 (3.09)
I3							
	009	Moderate	2.23 (0.67)	3.53 (2.05)	2.08 (3.16)	7.22 (2.99)	2.48 (3.29)
	010	Moderate	1.65 (0.92)	—^a^	—	7.95 (1.90)	2.47 (5.83)
	011	Mild	1.55 (0.23)	—	2.71 (2.20)	—	1.63 (1.28)

^a^Not available.

**Figure 4:**
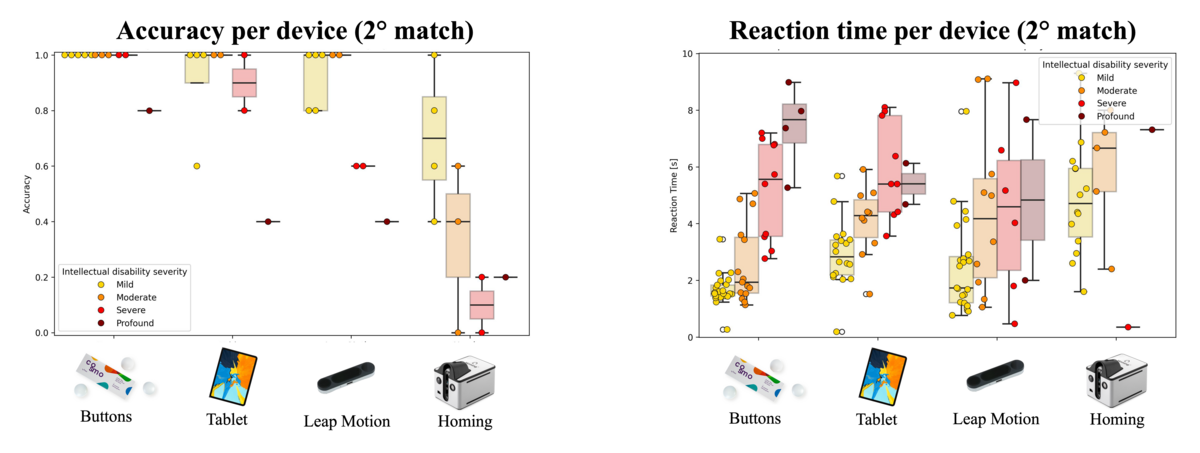
Boxplots representing the accuracy (left) and the reaction time (right) for each device and intellectual disability severity, considering only the second match played by each player per device in the Igloo minigame. Each point represents a single measurement.

At the device level, based only on the second match played by each participant, the buttons yielded the highest accuracy (median 1.00, IQR 0.00), followed by the tablet (median 1.00, IQR 0.20), the Leap Motion (median 0.90, IQR 0.35), and thus the Homing system (median 0.40, IQR 0.40). Reaction times increased from the buttons (median 1.89, IQR 2.09 seconds) to the Leap Motion (median 2.63, IQR 3.32 seconds), the tablet (median 3.63, IQR 2.25 seconds), and the Homing system (median 5.13, IQR 3.28 seconds). Figure 4 displays, for each device and intellectual disability severity, the boxplots of accuracy and reaction times considering only the second match played by each participant. For each device, a clear pattern emerges: as the level of intellectual disability severity increases, accuracy progressively decreases, while reaction times correspondingly increase.

The Spearman correlation between the intellectual disability severity and the overall median accuracy per participant resulted in a coefficient of –0.87 (95% CI –1.00 to –0.56) with a *P* value of <.001, indicating that as intellectual disability increases, the accuracy decreases. A significant positive association was also found between intellectual disability severity and overall reaction time per participant (ρ=0.69, 95% CI 0.08-0.94; *P*=.02), indicating that as intellectual disability increases, reaction times increase too.

##### Volcano Minigame

Table 7 displays for every participant the group to which they were assigned, their intellectual disability severity, the facilitation options used, their median accuracy for each device used, and their overall median accuracy.

**Figure 5:**
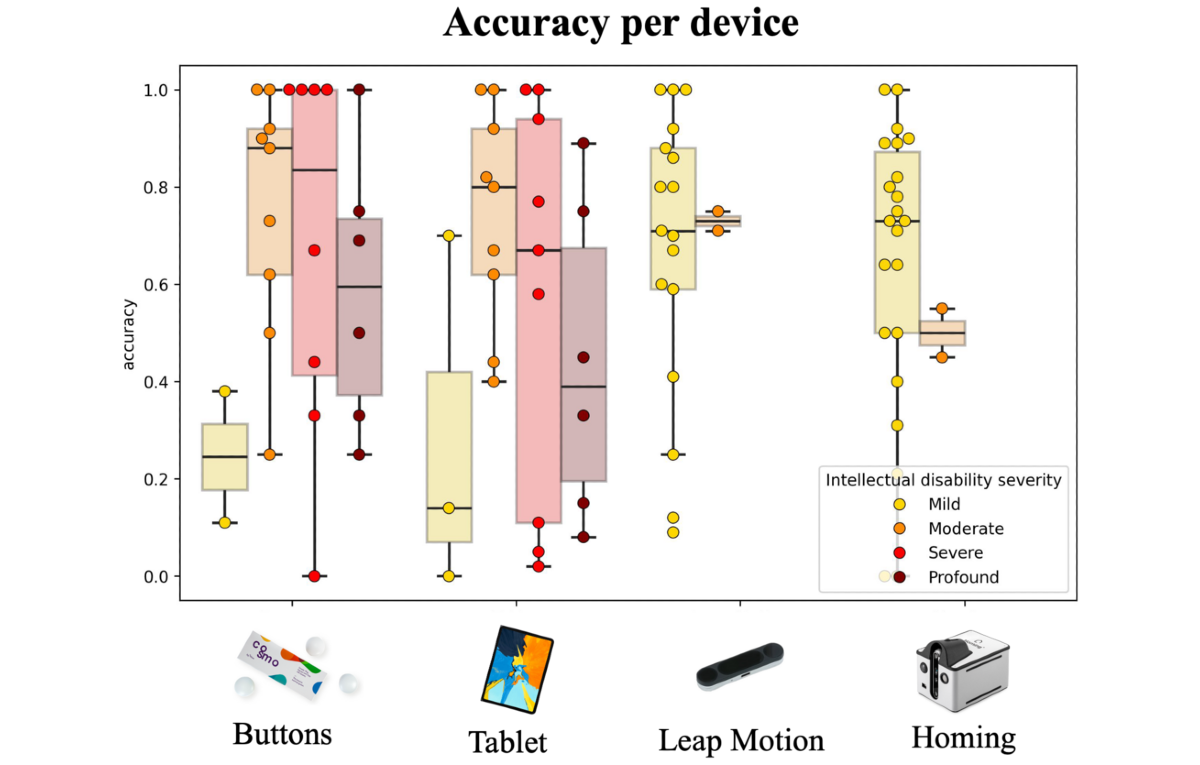
Boxplots representing the accuracy per device and intellectual disability severity in the Volcano minigame. Each point represents a single measurement.

Figure 5 shows boxplots of accuracy per device and intellectual disability severity. The only apparent exception concerns the mild category for the Buttons and tablet devices. However, as reported in Table 7, this result reflects data from a single participant who was assigned these devices based on availability within their group.

**Table 7:** Participants’ intellectual disability level and their Volcano minigame performance data.

Group	Participant	Intellectual disability severity	Facilitation options	Accuracy, median (IQR)				
				Buttons	Tablet	Leap Motion	Homing	Overall
V2	001	Severe	3D model, auto-go, no distracting stimuli, high contrast	1.0 (0.25)	0.68 (0.35)	N/A^a^	N/A	0.86 (0.54)
V3	002	Mild	—^b^	N/A	N/A	0.67 (0.00)	0.64 (0.31)	0.59 (0.42)
V3	003	Moderate	Clearer input, no distracting stimuli	0.62 (0.23)	0.53 (0.24)	N/A	N/A	0.62 (0.29)
V1	004	Profound	3D model, auto-go, no distracting stimuli, less movements	0.60 (0.36)	0.39 (0.48)	N/A	N/A	0.48 (0.44)
V2	005	Mild	No distracting stimuli	0.25 (0.14)	0.14 (0.35)	N/A	N/A	0.14 (0.27)
V2	006	Mild	High contrast	N/A	N/A	0.50 (0.09)	0.31 (0.43)	0.41 (0.36)
V3	007	Severe	3D model, no distracting stimuli	0.56 (0.34)	0.67 (0.95)	N/A	N/A	0.67 (0.67)
V1	008	Mild	—	N/A	N/A	0.86 (0.26)	0.79 (0.19)	0.82 (0.24)
V1	009	Moderate	Seated mode	N/A	N/A	0.73 (0.02)	0.50 (0.05)	0.63 (0.20)
V1	010	Moderate	No distracting stimuli	0.96 (0.09)	0.92 (0.20)	N/A	N/A	0.92 (0.12)
V3	011	Mild	No distracting stimuli	N/A	N/A	0.76 (0.11)	0.89 (0.17)	0.80 (0.18)

^a^N/A: not applicable.

^b^None.

**Figure 6:**
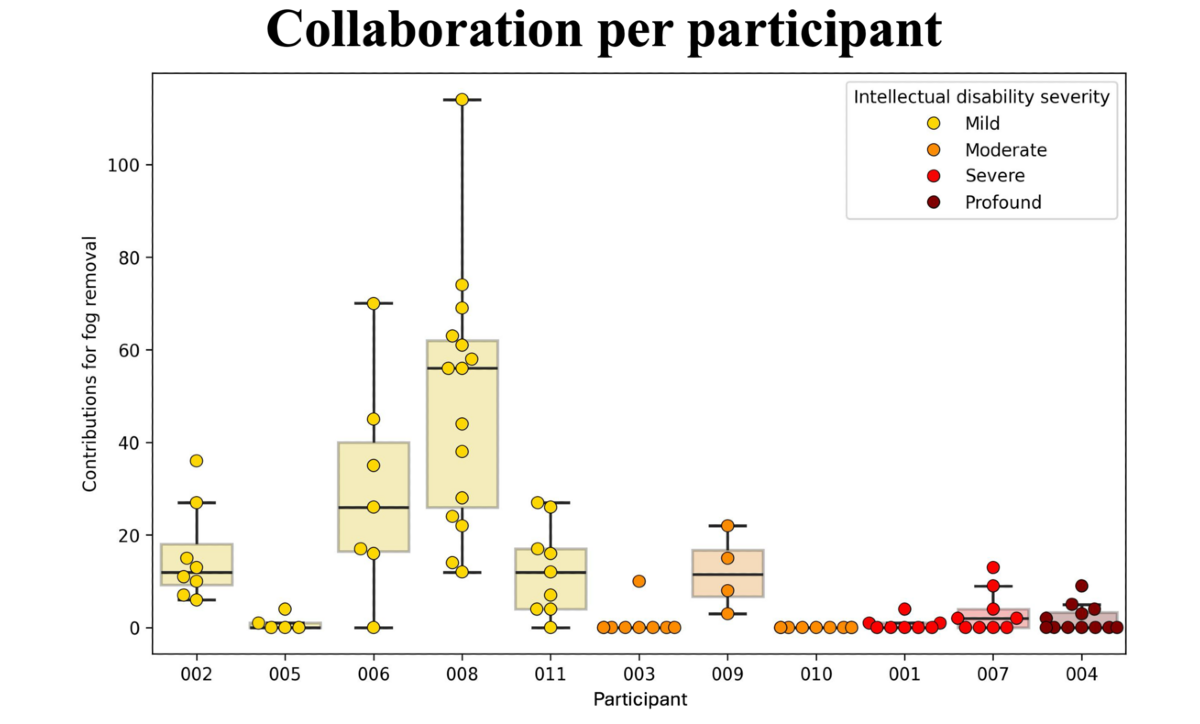
Boxplots representing the number of movements made by each participant to help remove the fog in the Volcano minigame. Each point represents a single measurement.

Figure 6 shows boxplots of the number of fog removal movements by individual user. Users are color-coded according to their intellectual disability severity level, with data pooled across all devices.

The Spearman correlation between intellectual disability severity and overall median accuracy yielded a *P* value of .48 (ρ=0.24, 95% CI –0.48 to 0.79), indicating no significant correlation was found. To verify that this observation was not driven by a single participant (ID 005), whose data represent the apparent exception among participants with mild intellectual disability severity, the analysis was repeated excluding their data, and the correlation remained nonsignificant (*P*=.82).

#### Behavioral, Satisfaction, and Usability Analysis

To analyze the types of behaviors observed during playtime, the total occurrences for each behavior category were obtained and presented in Table 8, divided per minigame.

Table 9 presents the median and IQRs for each question in the questionnaire completed by each operator at each game station, separately for both minigames.

Table 10 shows the medians and IQRs of the satisfaction and usability questionnaire for each question and minigame.

**Table 8:** Behaviors emerged during playtime for each minigame, divided into the 8 categories that emerged from the co-design phase.

	Positive verbal individual	Positive nonverbal individual	Positive verbal social	Positive nonverbal social	Negative verbal individual	Negative nonverbal individual	Negative verbal social	Negative nonverbal social
Igloo	100	22	0	1	1	0	0	0
Volcano	34	119	31	1	1	2	0	0

**Table 9:** Results of the questionnaire compiled by operators regarding the usability of the system by participants for each device and minigame^a^.

	Q1, median (IQR)		Q2, median (IQR)			Q3, median (IQR)	
	Igloo	Volcano	Igloo	Volcano	Igloo		Volcano
Buttons	5 (0)	4 (0)	5 (0.5)	3.25 (0.5)	5 (0.5)		3.25 (0.88)
Tablet	4 (1)	3.5 (0.38)	4 (2)	3 (0.38)	4 (2)		2.75 (0.88)
Leap Motion	5 (1.75)	5 (0.5)	5 (1.75)	4.5 (0.5)	5 (2)		4.5 (1.5)
Homing	4 (1)	4.5 (1)	3 (1.75)	4 (1)	3 (1.5)		3.5 (1)

^a^Questionnaire items Q4-Q6 assessed participants' overall experience during each game session rather than device-specific usability. The scores were as follows: Q4: median 4 (IQR 1.25) for Igloo and median 4 (IQR 0.25) for Volcano. Q5: median 3 (IQR 1) for Igloo and median 3.5 (IQR 1.5) for Volcano. Q6: not applicable for Igloo (this question did not apply to the Igloo minigame) and median 3.5 (IQR 2) for Volcano.

**Table 10:** Results of the satisfaction and usability questionnaire administered at the end of each session.

	Q1, median (IQR)	Q2, median (IQR)	Q3, median (IQR)	Q4, median (IQR)	Q5, median (IQR)	Q6, median (IQR)
Igloo	4.75 (0.88)	4 (0.38)	4 (0.5)	4 (0.75)	4.5 (0.5)	N/A^a^
Volcano	4.5 (1)	4 (0.5)	4 (0.5)	4 (0.88)	4.5 (0.75)	4 (0.75)

^a^N/A: not applicable.

## Discussion

### Principal Findings

This study aimed to co-design, develop, and evaluate Elemental, an innovative exergame that fully personalizes the gaming experience to accommodate each player’s needs, while still aiming to deliver an enjoyable and collaborative multiplayer experience. The evaluation of Elemental involved a group of individuals with IDDs and assessed usability, the impact of accessibility features on performance, and players’ satisfaction and enjoyment. The findings suggest that the co-design process led to the development of a feasible multiplayer exergame; that personalized devices and facilitation options reduced performance disparities associated with intellectual disability severity; and that participants reported positive usability, satisfaction, and enjoyment. Behavioral observations further suggested that the game supported collaboration during play. In line with the European Disability Strategy [3], this study supported that meaningful participation in digital gaming is possible, even for heterogeneous and demanding groups, when the design process is carried out with the participation of stakeholders and with the user in mind.

The co-design approach, based on the CeHRes Roadmap 2.0 [30], successfully identified the key challenge in group settings: accommodating diverse needs without creating separate experiences. This challenge is consistent with broader accessibility and inclusive design literature, which emphasizes that inclusion should be addressed at the level of design rather than through compensatory adaptations alone [12,13]. Unlike existing games that are either developed for neurotypical users and subsequently adapted or designed exclusively for people with disabilities [20], Elemental was conceived as inherently accessible. This addresses a persistent gap where specialized games often feel patronizing to neurotypical users, while mainstream games exclude people with disabilities [37]. Elemental was thus designed as a collaborative multiplayer exergame, offering all participants the same core experience, enriched by a customization layer that supports diverse needs without compromising the shared gameplay dynamics and overcoming traditional accessibility features. This solution enhances engagement and interaction among users with varied functional profiles, fostering meaningful cooperation. Unlike typical commercial or academic solutions that prescribe specific input devices for users with disabilities [16,17], the Elemental platform integrates multiple input modalities [2], ranging in complexity and interaction style, encouraging players to explore and engage in ways that suit their individual capabilities and preferences. This approach prioritizes user agency and enjoyment, rather than creating separate interaction pathways.

However, the main objective of this work was not only to develop an engaging game, but also to assess the impact of the aforementioned facilitation options. Elemental was thus developed as a modular platform, consisting, as of now, of 2 minigames: the Igloo and the Volcano. The first minigame’s purpose was to assess participants’ accuracy and reactiveness when playing with no facilitations and with the entire range of devices; the second minigame, on the contrary, was played with facilitations and with the best-performing devices, to assess accessibility features’ impact, enjoyment, and collaboration. The game was tested by a group of 11 adults with IDDs of varying severity, aged from 19 to 36 years. The initial interaction of participants with input devices was assessed through performance indicators of the Igloo minigame. Participants played with all input devices and no facilitation options, and the collected data revealed notable differences across devices. In the Igloo minigame, performance worsened as intellectual disability severity increased, indicating that even accessibility-focused games remain unequally usable when the experience and the interactions are not personalized, supporting the idea that traditional “one-size-fits-all” approaches systematically exclude people based on their functional limitations [38]. Despite participants with the most severe conditions being able to effectively use a limited number of devices, the others achieved good performance scores across all input methods.

Thus, to evaluate the accessibility potential of the exergame, in the Volcano minigame, players were assigned appropriate devices and played with tailored facilitation options, derived from the co-design phase to cover sensory, cognitive, and motor impairments, coherently with the main difficulties found in literature [21]. In the Volcano minigame, the absence of a significant association between disability severity and performance suggests that personalization can reduce performance disparities in heterogeneous groups. This result is likely linked to the combination of tailored facilitation options, assignment of more suitable devices, the group settings, familiarization effects, and specific game mechanics, such as the collaborative mechanics that allowed peers to support one another during play.

The heterogeneous grouping implemented in the Volcano minigame further facilitated peer support through verbal interactions, with behavioral data showing substantial verbal collaboration. This aligns with evidence suggesting that structured group activities can foster social participation and strengthen interpersonal skills in individuals with IDDs [6] and that meaningful engagement in shared leisure activities is associated with improved quality of life outcomes in this population [5]. Moreover, the high satisfaction scores and positive behavioral observations suggest that inclusive design does not compromise engagement. This is consistent with the broader literature on game-based approaches, which highlights how ludic elements can sustain motivation and active participation, particularly in populations that may otherwise struggle to engage with conventional activities [15]. Operators rated most aspects of usability highly, while participants reported enjoyment and willingness to play again. These findings challenge the paradigm commonly adopted when making the digital space accessible [21]. Rather than separate experiences or simplified gameplay, true accessibility emerged in a richer, more flexible environment where everyone’s contribution mattered.

### Limitations

This study had a few limitations. First, even though the questionnaires used to assess the opinions of operators and the usability and satisfaction of participants were adapted from existing questionnaires and classifications, they would require further validation and broader application to ensure their reliability and effectiveness. Additionally, due to unforeseen circumstances, not all participants were able to attend every scheduled session, and all sessions took place in the afternoon, potentially affecting participant performance due to fatigue. Another potential bias arose from logistical constraints in device availability, which occasionally required participants to use suboptimal configurations. However, this only affected individuals with lower-severity disabilities, and each case was closely monitored to ensure full participation and comfort.

Future studies should be conducted with a larger sample size, including both individuals with IDDs and neurotypical development, to better assess the game’s inclusivity, accessibility, and the effects of cooperation on players’ performance. Additionally, incorporating children in future research could help assess the game’s potential for this population.

Furthermore, it would be extremely interesting to assess whether playing the game could have some positive long-term impact on players’ lives. People with disabilities often have lower physical activity levels compared to the general population [18], and an engaging exergame such as Elemental could serve as a catalyst to increase habitual movement in this group, leveraging the motivational potential of play to overcome common barriers to physical activity. Similarly, further studies could organize longer and more frequent testing sessions, investigating whether some sort of training with this exergame can boost collaboration levels and reactiveness even outside of the game space.

### Conclusions

This study set out to co-design, develop, and evaluate the feasibility of Elemental, an inclusive multiplayer serious game for young adults with developmental disabilities. The findings indicate that, when an exergame is built through a structured co-design process, equipped with tailored features, and played under appropriate conditions, it can be both accessible and engaging for players with markedly diverse abilities and varying severity of intellectual impairment. The results further suggest that personalized devices and facilitation options can reduce performance disparities associated with intellectual disability severity. Beyond the specific game presented here, these results provide preliminary evidence for a design approach in which accessibility is embedded from the earliest stages of technology development rather than retrofitted through compensatory adaptations or separate, disability-specific solutions. Aligned with the social model of disability and the community-inclusion goals of the European Disability Strategy [3], such an approach has the potential to create meaningful opportunities for participation, social interaction, physical activity, and enjoyment for individuals who might otherwise encounter barriers to sharing activities with their peers. The same principles could support inclusive activities in educational and community-based environments, where adults with IDDs spend much of their time. Future studies with larger samples, ideally including both individuals with IDDs and neurotypical participants, are needed to confirm these findings, and longitudinal studies are required to evaluate their long-term benefits and impact. Ultimately, this work offers proof of concept for a fundamental shift: from asking how people with disabilities can be fitted into existing digital spaces, to asking how digital spaces can be designed to embrace diversity and to accommodate everyone’s needs.

## Data Availability

Due to ethical and legal considerations regarding the protection of participant privacy, the data supporting this study cannot be made available.

## Appendix

Multimedia Appendix 1 Images and descriptions of the facilitation options visible on screen.

Multimedia Appendix 2 Videos of the interaction of players with each device in each minigame.

## Abbreviations

ASPOC: Associazione per lo Sviluppo del Potenziale Cognitivo
CeHRes: Centre for eHealth and Wellbeing Research
IDD: intellectual and developmental disability
