# Supplementary material for: Inclusive and Collaborative Exergame for Adults With Intellectual and Developmental Disabilities: Development and Usability Study
Source: JMIR Serious Games. 2026 Jul 31;14:e90156. doi: 10.2196/90156 (PMC13476731; doi:10.2196/90156)
Supplement: Supplementary file 1 [file games_v14i1e90156_app1.pdf]

Multimedia Appendices 2

Chiara Piazzalunga[1]*, Samuele Ravazzani[1]*, Alberto Romano[2], Federica Maria Storti[1], Chiara Zelano[2], Emma Mencacci[2], Grazia Giana[2], Ottaviano Martinelli[2], Manuela Galli[1], Simona Ferrante[1,3]

1 Department of Electronics, Information and Bioengineering, Politecnico di Milano, Milan, Italy
2 Department of Mental Health and Addiction, Alessandro Manzoni Hospital ASST Lecco, Lecco, Italy
3 LEARNLAB, Joint Research Platform, Fondazione IRCCS Istituto Neurologico Carlo Besta, Milan, Italy

* These authors contributed equally to the study.
Corresponding author: Samuele Ravazzani, samuele.ravazzani@polimi.it

Facilitation options

Increased contrast

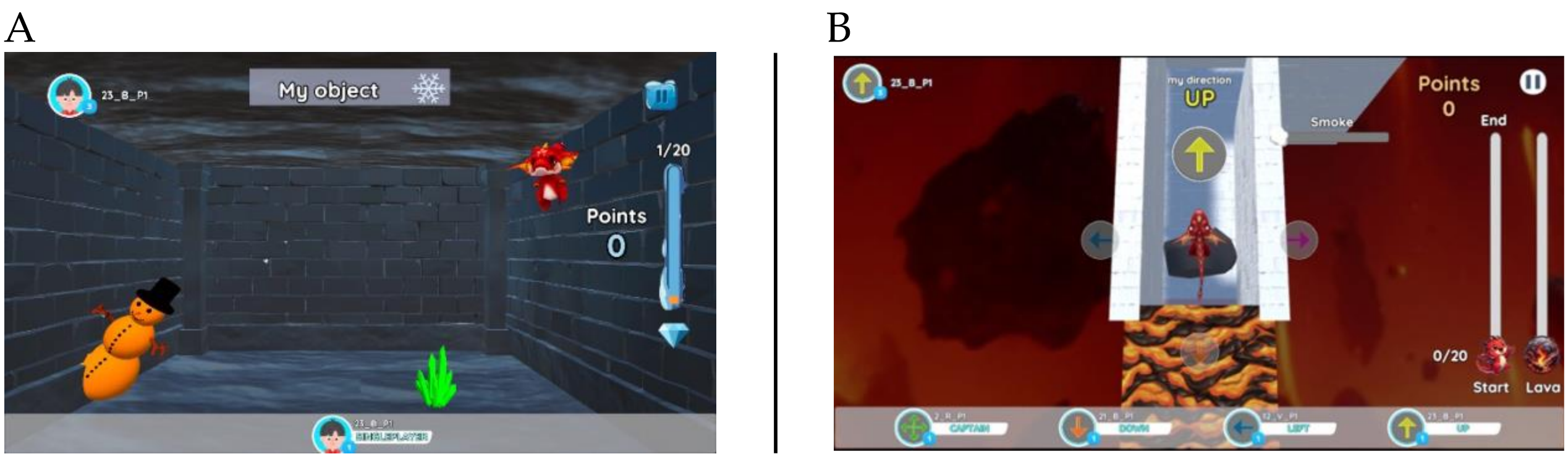

Figure 1. Increased contrast facilitation option for Igloo (A) and Volcano (B)

With the increased contrast facilitation option, in both minigames the background appears darker (Figure 1). In the Igloo objects have different colours to appear more evident, while in the Volcano walls are white to better result from the red of the background.

Clearer input

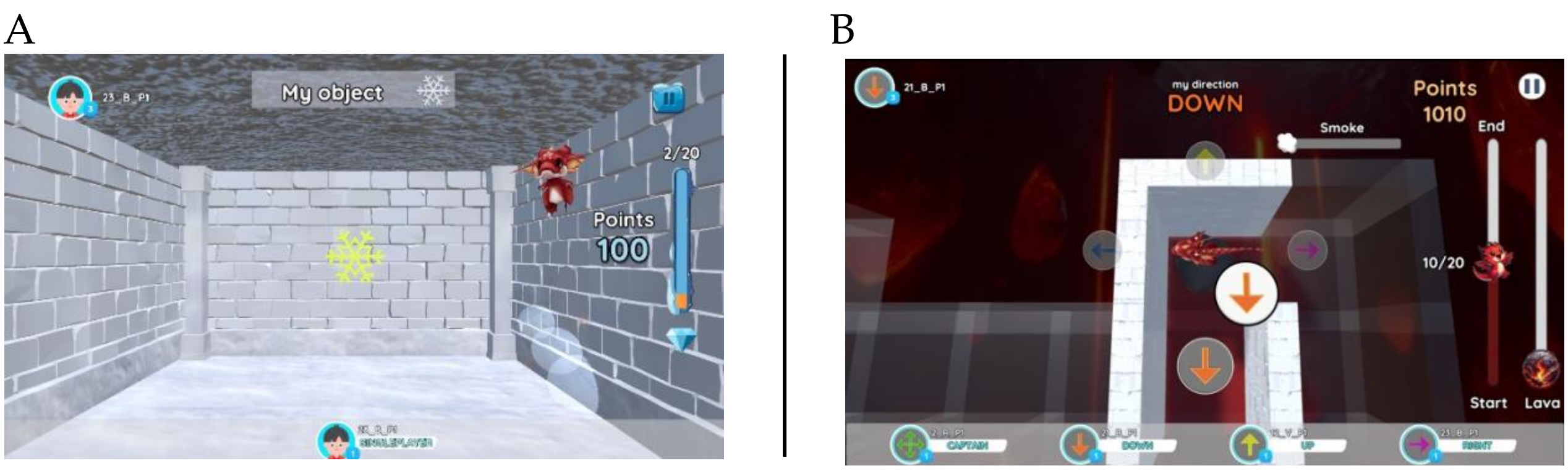

Figure 2. Clearer input facilitation option for Igloo (A) and Volcano (B)

With the clearer input facilitation option (Figure 2), in the Igloo the stimuli of other players appear as semi-transparent, while those of the actual player appear completely visible. In the Volcano, walls of

the cells of the other players appear semi-transparent, while those to which the subject should react appear as white, and an additional icon with white background appears.

3D model advisor for Cosmo Switch buttons

A B

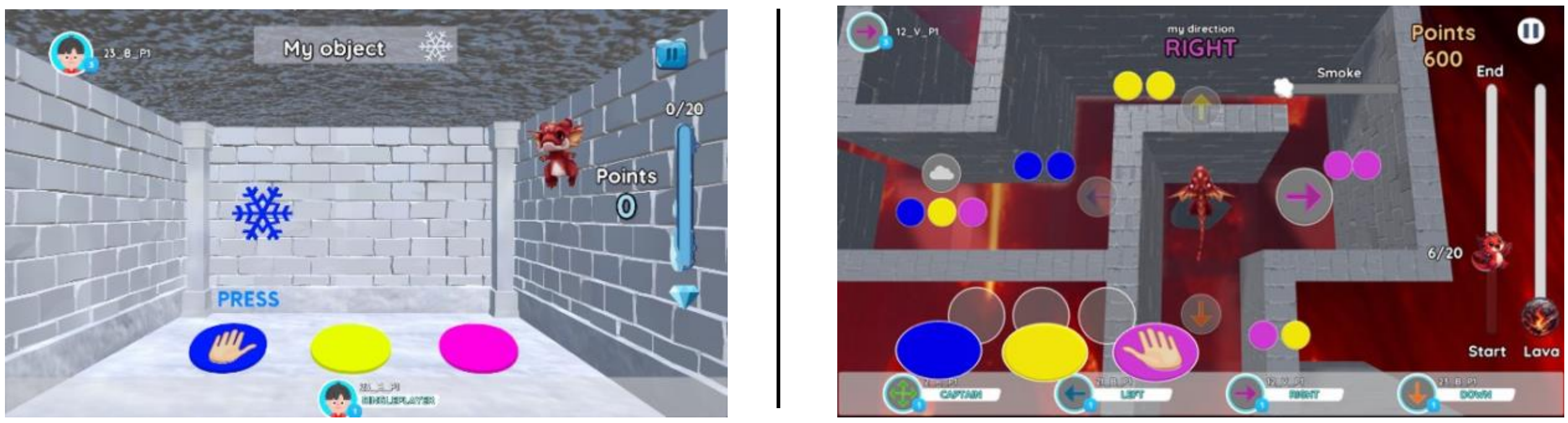

Figure 3. 3D model advisor for Cosmo Sqitch buttons for Igloo (A) and Volcano (B)

With the 3D model advisor facilitation option for Cosmo Switch buttons (Figure 3), in both minigames a virtual representation of the buttons appears, with virtual hands simulating the movement when pressing.

3D model advisor for tablet

A B

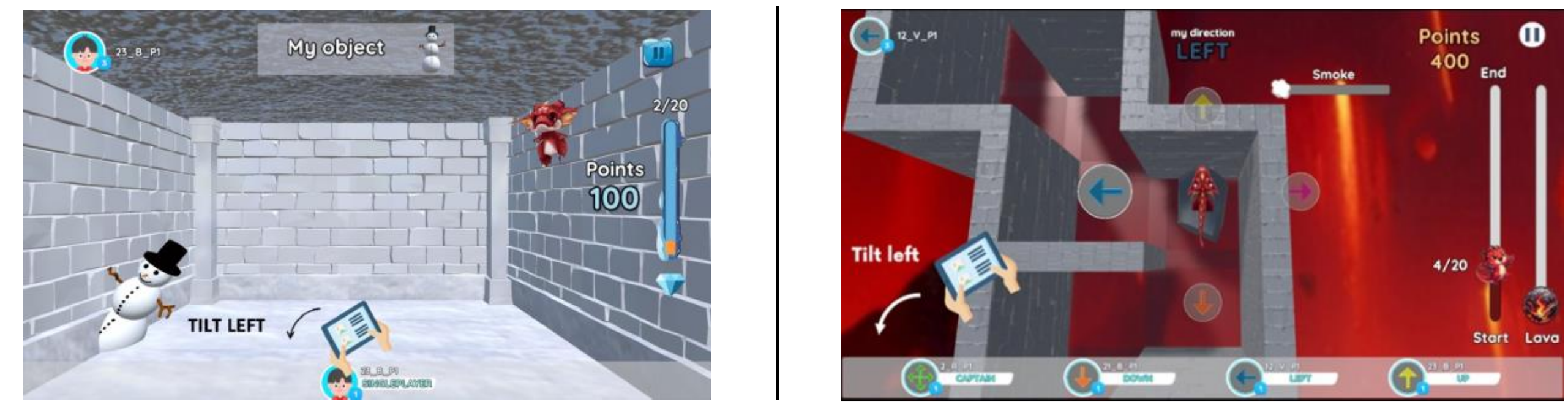

Figure 4. 3D model advisor for tablet for Igloo (A) and Volcano (B)

With the 3D model advisor facilitation option for tablet (Figure 4), in both minigames a virtual representation of the tablet hold by hands appears and simulates a tilting movement.

3D model advisor for Leap Motion Controller

A B

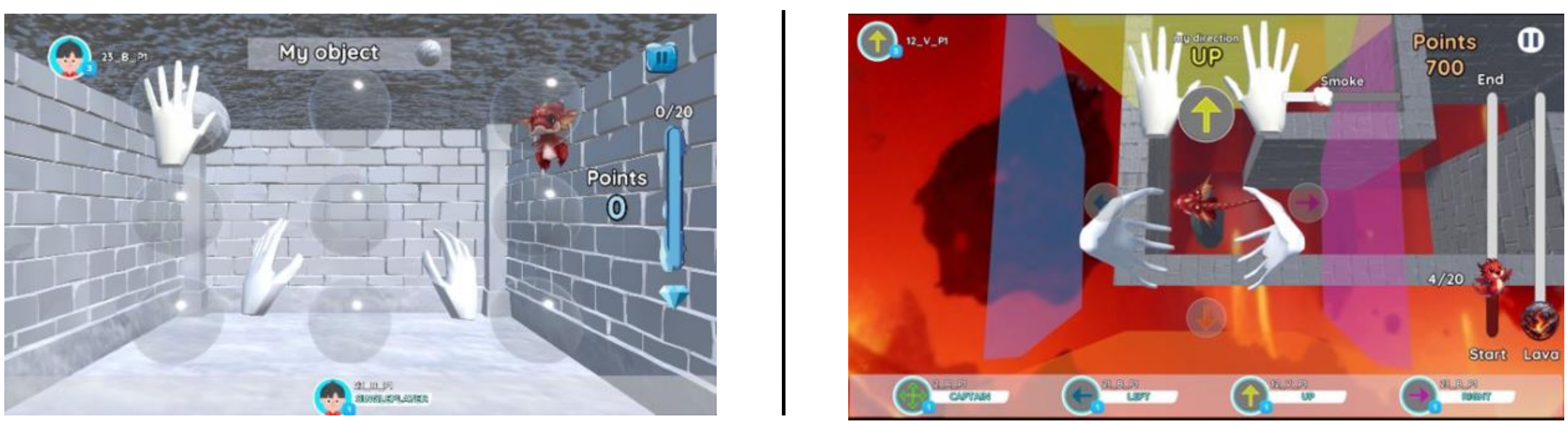

Figure 5. 3D model advisor for Leap Motion Controller for Igloo (A) and Volcano (B)

With the 3D model advisor facilitation option for Leap Motion Controller (Figure 5), in both minigames an additional virtual representation of the hand appears, indicating where virtual hands should go.

3D model advisor for Homing

A B

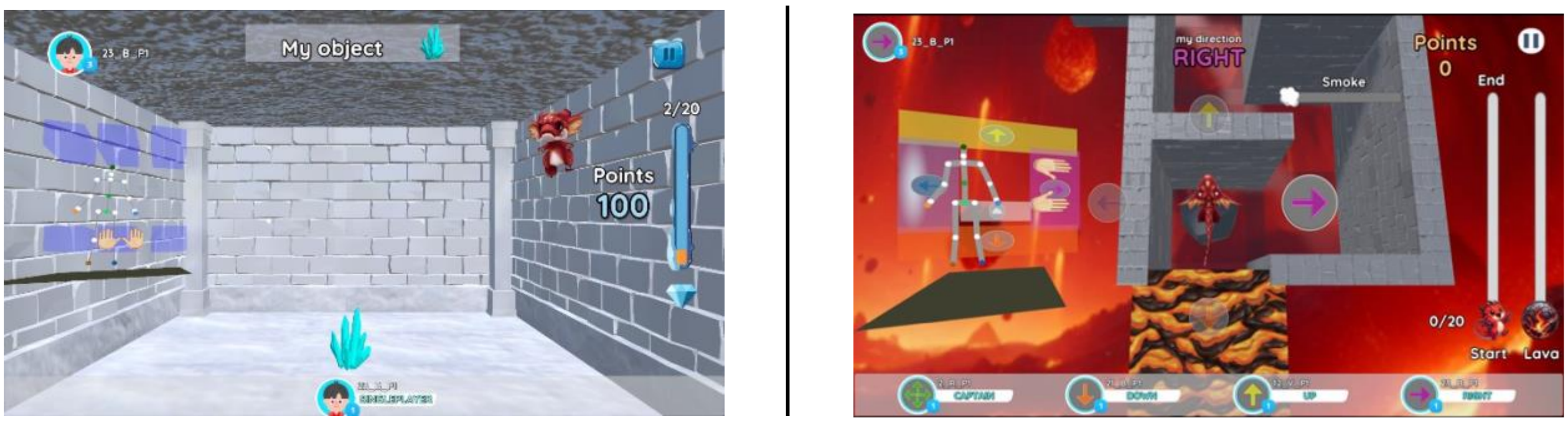

Figure 6. 3D model advisor for Homing for Igloo (A) and Volcano (B)

With the 3D model advisor facilitation option for Leap Motion Controller (Figure 6), in both minigames a virtual representation of the hands appears, indicating where virtual hands should go.
